# The One Big Beautiful Bill: A Looming Crisis for Health Equity and Emergency Medicine

**DOI:** 10.5811/westjem.52943

**Published:** 2025-12-20

**Authors:** Melanie Yates, Peter Yun

**Affiliations:** * George Washington University, School of Medicine and Health Sciences, Department of Emergency Medicine, Washington, DC; † University of Texas Southwestern Medical Center, Department of Emergency Medicine, Dallas, Texas

On July 4, 2025, the budget reconciliation bill—colloquially known as the One Big Beautiful Bill Act (OBBBA)—was signed into law, marking the first step in a slew of sweeping reforms across Medicaid, Medicare, and the Patient Protection and Affordable Care Act marketplaces.^1^ While the legislation was promoted as a bold fiscal restructuring, its healthcare provisions carry profound implications for emergency medicine. For many Americans, the OBBBA is not an abstract budgetary exercise but a lived reality that will reshape how, when, and whether they can access care.

At the heart of the law are several major Medicaid changes: the introduction of nationwide work requirements, tighter and more frequent eligibility redeterminations, restrictions on immigrant eligibility, and new limits on state Medicaid financing flexibility.^2^ According to the Congressional Budget Office, the law will reduce federal spending on healthcare by over $1 trillion and lead to an increase of over 10 million uninsured individuals.^1^ For those of us who work in the emergency department (ED), the consequences will be immediate and visible: more patients arriving uninsured, sicker, and later in the course of their illness.

Emergency physicians often highlight the 1986 Emergency Medical Treatment and Labor Act (EMTALA), one of the most comprehensive laws granting nondiscriminatory access to emergency medical care, as a defining aspect of our specialty. This law guarantees that no patient will ever be turned away, regardless of ability to pay, race, sex/gender, background, or creed.^3^ But EMTALA is an unfunded, safety-net mandate, not a substitute for comprehensive coverage, and its interplay and continued efficacy as health policy law continues to change.^4^ The law was designed to prevent patient dumping and was the nation’s promise to patients of access to a doorway, but it does not guarantee desperately needed access to longitudinal or preventative care that actually save lives.^5^

The OBBBA threatens to expose the fragility of relying on EMTALA alone as the medical care safety net of this nation. The Congressional Budget Office estimates that the work requirement provisions alone will reduce federal Medicaid spending by $326 billion over 10 years and will lead to 5.3 million more uninsured patients by 2034.^1^ This change will increase uncompensated care and widen health inequities. A major study from the National Bureau of Economic Research found that Medicaid expansion in certain states under the Affordable Care Act reduced the overall risk of death by 2.5%, and by as much as 20% among those newly eligible. Medicaid expansion saved approximately 27,400 lives between 2010 and 2022.^6^ Similarly, it was calculated that Medicaid expansion was associated with a net decrease of 31.8 deaths per 100,00 person-years following the COVID-19 pandemic. This trend was statistically significant for deaths related to chronic disease, and the protective effect of Medicaid was present regardless of age, sex, or race.^7^ Without Medicaid, we will be working against these positive trends.

Not only does the OBBBA target the recipients of Medicaid and their eligibility for the program, it significantly diminishes funding for the program via cap reductions in the Medicaid provider taxes from 6% to 3.5%. Rural hospitals disproportionately rely on Medicaid to keep the lights on, and the cap reductions caused so much alarm in states with higher Medicaid populations^8^ that the Rural Health Transformation Program (RHTP) was added to assuage policymakers with largely rural districts to sign the OBBBA. The RHTP is a $50 billion fund administered through the Centers for Medicare & Medicaid over five years, advertised as a way to offset funding decreases from Medicaid. However, based on the way the RHTP is currently written, it is unlikely to make up for the substantial Medicaid cuts, especially with no guarantees that the funding will reach rural hospitals and their patients.^9^

Rural hospitals are not the only hospitals at risk. Urban safety-net EDs, particularly those in academic medical centers, will shoulder worsening disproportionate strain. According to the Association of American Medical Colleges (AAMC), while its members comprise approximately 5% of hospitals in the United States, AAMC member centers deliver about 32% of all uncompensated care in the US.^10^

The financial stress created by the OBBBA on both rural and academic hospitals will accelerate closures and cause significant strain on their EDs. Without access to primary clinics for preventative care, patients will arrive at the door of the ED to access unscheduled and often uncompensated care, exacerbating EDs’ crowding and boarding problems. More crowded EDs mean longer wait times, higher left-without-being-seen rates, increased boarding, and poorer patient outcomes. With the burnout rate already so high in emergency medicine, the potential worsening moral burden of emergency physicians—who must care for patients in increasingly inequitable and under-resourced circumstances—cannot be understated.^11^

As a specialty, emergency medicine must remain vigilant. The passage of the OBBBA represents not just a fiscal shift but a public health turning point, much as the Affordable Care Act was several years ago. Regardless of one’s political stance, the reality of this bill is clear: Millions will lose coverage, and the downstream effects will arrive at the doorsteps of EDs first. We must advocate for policies that preserve equitable access to care, anticipate the resource challenges ahead, and reject the illusion that EMTALA alone can sustain the nation’s health.

This means continuing our own education, teaching residents the fundamentals of health policy (a recognized American Accreditation Council for Graduate Medical Education core competency), advocating through professional organizations, and working with local hospital leadership. The system for implementing the Medicaid work requirements will be state based; so there is still time for advocacy to key stakeholders and leaders to improve the ease of reporting requirements and reduce the inappropriate loss of insurance coverage. Rural and underserved hospitals with high numbers of uninsured and Medicaid populations are at greatest risk for lost funding and closure; urging state and federal policymakers to equitably apply and disperse the RHTP funds will be key to reducing these inequities.

These policy changes also present a unique opportunity for emergency physicians and departments to develop partnerships with community organizations to bridge the gaps in access and improve health equity. Many community and philanthropic organizations that have been filling the gaps and needs for many patients will face more barriers as need grows and funding options diminish. Strengthening partnerships and supporting them to continue their critical work will be especially important. Additionally, the private sector holds significant potential to fund innovations in healthcare access, such as in frontiers of telehealth or artificial intelligence that may decrease healthcare inequalities and reduce emergency physician workload and burnout.^12^,^13^ If we truly want to save lives, we must lead the effort to build and sustain the infrastructure required to do so. Now is the time to advocate, speak up, and get ready.

Emergency medicine has always been proud to be the last line of defense for our safety net. Without broader systemic investment in access and equity, EMTALA risks becoming not a net but a thin thread—one that risks breaking under the weight of continued policy changes that limit access to healthcare.
